# Minnesota Impulse Disorders Interview (MIDI): Validation of a structured diagnostic clinical interview for impulse control disorders in an enriched community sample

**DOI:** 10.1016/j.psychres.2018.05.006

**Published:** 2018-07

**Authors:** Samuel R. Chamberlain, Jon E. Grant

**Affiliations:** a Department of Psychiatry, University of Cambridge, UK; b Cambridge and Peterborough NHS Foundation Trust (CPFT), UK; c Department of Psychiatry & Behavioral Neuroscience, University of Chicago, Pritzker School of Medicine, 5841 S. Maryland Avenue, MC 3077, Chicago, IL 60637, USA

## Abstract

**Background:**

Disorders of impulsivity are common, functionally impairing, and highly relevant across different clinical and research settings. Few structured clinical interviews for the identification and diagnosis of impulse control disorders exist, and none have been validated in a community sample in terms of psychometric properties.

**Methods:**

The Minnesota Impulse control disorders Interview (MIDI v2.0) was administered to an enriched sample of 293 non-treatment seeking adults aged 18–35 years, recruited using media advertisements in two large US cities. In addition to the MIDI, participants undertook extended clinical interview for other mental disorders, the Barratt impulsiveness questionnaire, and the Padua obsessive-compulsive inventory. The psychometric properties of the MIDI were characterized.

**Results:**

In logistic regression, the MIDI showed good concurrent validity against the reference measures (versus gambling disorder interview, *p *< 0.001; Barratt impulsiveness attentional and non-planning scores *p *< 0.05), and good discriminant validity versus primarily non-impulsive symptoms, including against anxiety, depression, and obsessive-compulsive symptoms (all *p *> 0.05). Test re-test reliability was excellent (0.95).

**Conclusions:**

The MIDI has good psychometric properties and thus may be a valuable interview tool for clinical and research studies involving impulse control disorders. Further research is needed to better understanding the optimal diagnostic classification and neurobiology of these neglected disorders.

## Highlights

• Few structured clinical interviews for the identification of impulse control disorders exist.
• Of 293 adults in a community sample, 32.8% had at least one impulse control disorder, with gambling disorder, skin picking disorder, and compulsive buying being the most common.
• The Minnesota Impulse Disorders Interview showed good convergent and discriminant validity, and excellent test re-test reliability.

## 1 Introduction

Impulsivity refers to a multitude of behaviors that are poorly thought out, hasty, risky, and that lead to untoward functional consequences (Evenden, 1999, Stanford et al., 2016). Examples of impulse control disorders include gambling disorder, trichotillomania (hair pulling disorder), kleptomania (stealing), pyromania (fire-setting), intermittent explosive disorder, compulsive buying, compulsive sexual behavior, binge-eating disorder, and skin-picking disorder (Grant, 2008, McElroy et al., 1995). Many of these conditions were listed together under the category of impulse control disorders in DSM-IV and other disorders are regarded as impulsive due to overlap with other more classic impulse disorders (e.g. in terms of comorbidity and neurobiology) (Grant et al., 2014). In DSM-5, Gambling Disorder was reclassified as a Substance-Related and Addictive Disorder, whereas trichotillomania and skin picking disorder are now considered Obsessive-Compulsive Related Disorders. Binge-eating Disorder is listed as a Feeding and Eating Disorder. For the purposes of this paper, we consider these disorders collectively as impulse control disorders for convenience (and since they are often comorbid) but due attention should also be given to DSM-5 categories when assessing patients. Other disorders of course have impulsive symptoms, such as manic symptoms in Bipolar Disorder, or impulsivity in attention-deficit hyperactivity disorder (ADHD). We did not include mood disorders and ADHD in the MIDI because we felt that instruments already exist for their identification and diagnosis, and we wished the MIDI to focus on the other disorders in the interests of avoiding an excessively long interview. The impulse control disorders in the MIDI each have prevalence rates of around 0.5–5% in community settings, although gambling disorder may be considerably higher (Grant et al., 2014, Nowak and Aloe, 2014). The occurrence of impulse control disorders can also be markedly higher in clinical settings, such as in psychiatric inpatients (Grant et al., 2005), or in patients with Parkinson's Disease treated with dopamine agonists (Zurowski and O'Brien, 2015). Studies have shown that impulse control disorders are associated with pronounced functional impairments across a range of domains (e.g. Black, 1996, Grant and Kim, 2005, Grant et al., 2016, Phu et al., 2014).

Despite the considerable clinical importance and functional impact of impulse control disorders, there are few if any validated standardized interviews suitable for assessing and diagnosing these conditions in the round. The few existing instruments were designed for particular patient populations [such as for use in Parkinson's Disease (Weintraub et al., 2009)], for diagnosing impulse disorder rather than the range of them (Grant et al., 2004), or have not been validated in a community sample (Grant, 2008). In particular, the Minnesota Impulse control disorders Interview (MIDI) was originally developed in 2008 for the diagnosis of compulsive buying, kleptomania, trichotillomania, intermittent explosive disorder, pyromania, pathological gambling (now known as gambling disorder), and compulsive sexual behavior (Grant, 2008). The instrument showed good classification accuracy compared to detailed interviews by experienced psychiatrists in an inpatient setting, and also found impulse control disorders to be common in psychiatric inpatients (Grant et al., 2005). Building on this initial groundwork, the aims of the current study were (i) to update the MIDI to include other impulse control disorders (binge-eating disorder, skin-picking disorder), and to be consistent with DSM-5 diagnostic criteria for specific disorders; and (ii) to confirm its psychometric properties in an enriched community sample.

## 2 Methods

### 2.1 Participants

Advertisements were placed in two large US cities, asking for people to take part in a research study about impulsive behaviors. The advertisements were placed in a variety of locations including local newspapers, and bill boards. Data were collected from June 2013 to July 2016. This was an enriched sample in that we expected the occurrence of impulse control disorders to be elevated due to the nature of the advertisement. Adults aged 18–29 years were entered into the study after providing written informed consent. We focused on this age range because the MIDI was originally developed for use in adults, and impulse control disorders have peak onset in adolescence (Kessler et al., 2007) and thus are expected to subtend a major burden in young adults. Inability to understand/undertake the procedures was exclusionary. Participants attended the research center twice at least three months apart, and received a $50 gift card. The study procedures were carried out in accordance with the most recent version of the Declaration of Helsinki. The Institutional Review Board of the University of Chicago approved the study and the informed consent procedures.

### 2.2 Assessments

The original version of the Minnesota Impulse control disorders Interview (MIDI) (Grant, 2008) was revised to make diagnostic criteria for gambling disorder consistent with the latest version of the Diagnostic and Statistical Manual Version 5 (DSM-5) (American Psychiatric Association, 2013); furthermore, binge-eating disorder and skin picking disorder were also added (these disorders are recently newly recognized in DSM-5). The MIDI v2.0 is included in Appendix A. On the MIDI, subjects first respond to a general question about the given disorder; if responding affirmatively, the clinical interview then asks about other areas based on diagnostic criteria. Positive response to all questions indicates presence of a given impulse disorder, except for gambling disorder where endorsement of 5 or more items is required.

Each participant attended the study center to complete an extended clinical interview with a trained rater, in addition to completing self-report questionnaires. Baseline demographic information was recorded including age, gender, and level of education. Clinical assessment comprised the MIDI v2.0, the Structured Clinical Interview for Gambling Disorder (SCI-GD) (Grant et al., 2004), the Mini International Neuropsychiatric Inventory (MINI) (Sheehan et al., 1998), the Hamilton Depression Rating Scale (HAM-D) (Hamilton, 1960), and the Hamilton Anxiety Rating Scale (HAM-A) (Hamilton, 1959). The SCI-GD is a structured clinical interview based on DSM-5 criteria for gambling disorder (4 or more symptoms for diagnosis) updated from an earlier validated scale that used DSM-IV criteria (Grant et al., 2004). Note that the original validation paper for the SCI-GD differs in that the current version was adapted to be consistent with DSM-5 versus DSM-IV, by removing the gambling item “illegal acts” . The MINI is a structured clinical interview based on DSM criteria, which identifies mainstream mental disorders including mood, anxiety, and psychotic disorders (Sheehan et al., 1998). The HAM-D and HAM-A are widely used clinical scales for assessing severity of depressive and anxiety symptoms respectively (Hamilton, 1960, Hamilton, 1960).

Self-complete questionnaires included the Quality of Life Inventory (QOLI) (Frisch et al., 1992), the Barratt Impulsiveness questionnaire (version 11) (Barratt, 1965, Stanford et al., 2016), and the Padua obsessive-compulsive inventory revised (Burns et al., 1996, Sanavio, 1988). The QOLI provides an overall t-score corresponding to overall quality of life for the given individual, taking into account multiple important domains of functioning. The Barratt questionnaire (version 11) is 30-item questionnaire capturing various aspects of impulsivity, which yields three factor scores: motor impulsiveness, non-planning impulsiveness, and attentional impulsiveness (Patton et al., 1995). The Padua inventory revised is a 39-item questionnaire designed to measure obsessive-compulsive symptoms, both in normative populations and in OCD (Burns et al., 1996, Sanavio, 1988). Our rationale for including these two scales was to confirm that the MIDI loaded onto impulsive (Barratt) measures but not obsessive-compulsive symptoms (Padua inventory) as part of confirmation of its concurrent and discriminant validity respectively.

### 2.3 Data analysis

The overall baseline demographic and clinical characteristics of the sample were presented in summary form, including the frequency of impulse control disorders. We used binary logistic regression (maximum likelihood, logit) in JMP Pro to identify baseline demographic and clinical variables associated with positive screening for one or more impulse control disorders on the MIDI (binary variable). The following variables were included in the model: age, gender, education level, number of gambling disorder criteria met on the SCI-GD, presence of any mood disorder, presence of any anxiety disorder, depression scores (HAM-D), anxiety scores (HAM-A), Barratt motor/attentional/non-planning impulsiveness scores, and Padua obsessive-compulsive total scores. All variables were treated equally within the model i.e. on the same conceptual plane for simplicity. To confirm concurrent validity of the model, we expected SCI-GD and Barratt impulsiveness scores to be significant contributors to the model. To confirm discriminant validity of the scale against primarily non-impulsive symptoms, we expected occurrence of mood disorders, occurrence of anxiety disorders, state depression and anxiety scores, and OCD symptoms (Padua inventory) to be relatively weak contributors to the model.

The sensitivity and specificity of the MIDI for diagnosing impulse control disorders were computed against an interview for diagnosing gambling disorder, namely the Structured Clinical Interview for Gambling Disorder (SCI-GD) updated from a previously validated (Grant et al., 2004), adapted for DSM-5 (since the original instrument was for DSM-IV criteria). Sensitivity and specificity against other impulse control disorders was not possible because (i) the prevalence of other impulse control disorders was relatively low; and (ii) there exist no gold-standard diagnostic instruments for the other impulse control disorders. Test re-test for the MIDI was quantified in terms of concordance of presence or absence of impulse control disorders when participants were re-tested approximately 3 months after the baseline assessment.

## 3 Results

The sample comprised 293 adults (Table 1), of whom 96 [32.8%] had one or more impulse control disorders on the MIDI. The percentages of subjects in the sample with specific impulse control disorders were, in order of decreasing percentage: gambling disorder 81 [27.6%], skin picking disorder 17 [5.8%], compulsive buying 16 [5.5%], compulsive sexual behavior 10 [3.4%], intermittent explosive disorder 8 [2.7%], binge-eating disorder 8 [2.7%], kleptomania 2 [0.7%], and pyromania 2 [0.7%], and trichotillomania 1 [0.3%].

**Table 1:** Baseline demographic and clinical characteristics of the sample (*N *= 293).

Demographic/Clinical Measure	Mean (Standard Deviation) or *N* [% of sample]
Age, years	22.5 (3.8)
Gender, male	179 [61.1%]
Education level #	3.2 (0.8)
SCI-GD, number of criteria met (out of 9)	1.7 (2.3)
HAM-D	5.6 (5.9)
HAM-A	6.1 (6.1)
Barratt attentional impulsiveness	17.2 (4.2)
Barratt motor impulsiveness	24.2 (5.1)
Barratt non-planning impulsiveness	24.3 (5.3)
Padua obsessive-compulsive symptom score	23.6 (56.0)
Presence of one or more MIDI impulse control disorder	96 [32.8%]
Presence of mood disorder on the MINI	17 [5.8%]
Presence of anxiety disorder on the MINI	47 [16.0%]
Presence of psychosis on the MINI	0 [0%]

SCI-GD: Structured Clinical Interview for Gambling Disorder; HAM-D: Hamilton depression scale; HAM-A: Hamilton anxiety scale; MIDI: Minnesota Impulse Disorder Interview; MIDI: Mini International Neuropsychiatric Inventory. # education score definitions were: 1 = less than high school, 2 = high school graduate, 3 = some college education, 4 = college graduate, 5 = higher than college level education.

The regression model was significantly predictive of occurrence of one or more MIDI impulse control disorders (Chi-square [df = 12] = 66.51, p < 0.001). The parameter estimates and statistical significance of predictor variables are shown in Table 2. Subsequent interpretations all refer to findings once other variables were accounted for (due to the nature of the statistical model). Age, education level, and gender were not significantly associated with presence of an impulse disorder on the MIDI. The concurrent validity of the MIDI was confirmed, with SCI-GD (*p *< 0.001) and impulsiveness scores (*p *< 0.05 on Barratt attentional and non-planning subscores) being significant predictors of positive MIDI classification. The discriminant validity of the MIDI against non-impulsive disorders (i.e. disorders not primarily impulsive in nature) was confirmed, with relatively weak (and non-significant) associations being found between positive MIDI screen and mood/anxiety indices, and compulsivity (Padua inventory) scores (all *p *> 0.05). As expected, the presence of one or more impulsive disorders on the MIDI was associated with significantly lower quality of life on the Sheehan Quality of Life (41.6 [13.0] versus 46.8 [11.5]; ANOVA *F [df 1,280] *= 10.219, *p *= 0.001). The sensitivity of the MIDI against the sample gold-standard diagnosis for Gambling Disorder was 86.3% and the specificity was 84.7%. The test re-test reliability of the MIDI was 0.95 (excellent).

**Table 2:** Results of regression model showing relationships between baseline demographic/clinical characteristics and positive screen for one or more impulse control disorders on the MIDI. The overall model was significant (*p *< 0.001).

Variable	Estimate	Std. Error.	95% CI FOR estimate		L-R Chi-Sq	P value
			Lower CI	Upper CI		
Age	0.236664	0.148521	−0.03978	0.550441	2.782613	0.0953
Education level	0.222333	0.38178	−0.51026	1.009386	0.345793	0.5565
Gender	−0.59819	0.353736	−1.34797	0.062605	3.127321	0.077
SCI-GD total number of criteria met	0.591585	0.148657	0.327004	0.9195	22.27766	**<0.0001**
MINI, presence of current mood disorder	−1.05081	1.344862	−3.85283	1.490465	0.63349	0.4261
MINI, presence of current anxiety disorder	−1.36687	0.903898	−3.27055	0.309982	2.506386	0.1134
HAM-D total score	0.155334	0.092064	−0.0195	0.34671	3.019149	0.0823
HAM-A total score	−0.01867	0.078684	−0.1798	0.138805	0.056399	0.8123
Barratt impulsiveness, attentional	0.225675	0.117326	0.008427	0.476877	4.158316	**0.0414**
Barratt impulsiveness, motor	−0.05332	0.091755	−0.23772	0.127627	0.33848	0.5607
Barratt impulsiveness, non-planning	0.175744	0.093287	0.382442	0.010389	4.388758	**0.0362**
Obsessive-compulsive symptoms (Padua inventory) total score	−0.00348	0.011165	−0.03009	0.005423	0.171468	0.6788

SCI-GD: Structured Clinical Interview for Gambling Disorder; MINI = Mini International Neuropsychiatric Inventory; HAM-D Hamilton depression scale; HAM-A Hamilton anxiety scale.

## 4 Discussion

In this study, the psychometric properties of the Minnesota Impulse Disorder Interview (MIDI v2.0) were characterized in 293 non-treatment seeking adults recruited from the general community. We updated the original version of the MIDI (Grant, 2008) to make gambling disorder criteria consistent with DSM-5 criteria, and to include two additional disorders now recognized in the DSM (skin-picking disorder and binge-eating disorder) (American Psychiatric Association, 2013). The overall finding was that the MIDI 2.0 had good psychometric properties including high concurrent validity (against other instruments for assessing these disorders), good discriminant validity (relatively weak and non-significant associations with primarily non-impulsive symptoms), and high test-retest validity. Compared to a gold standard diagnostic interview for gambling disorder, the MIDI also exhibited good sensitivity and specificity.

Psychometric properties of the scale were evaluated using regression. The extent of gambling disorder symptoms on a gold-standard instrument (Structured Clinical Interview for Gambling Disorder, SCI-GD), and of personality-related impulsivity (Barratt questionnaire) were both significantly associated with the presence of impulse control disorders on the MIDI (*p *< 0.001 for SCI-GD, *p *< 0.05 for Barratt attentional and non-planning scores). These results support the concurrent validity of the scale. The divergent validity of the MIDI was also confirmed, by demonstrating non-significant associations between presence of impulse disorder on the MIDI and other clinical measures, including presence of formal mood or anxiety disorder (both *p *> 0.10), state depression and anxiety scores (HAM-D and HAM-A) (both *p *> 0.05), and obsessive-compulsive dimensional symptoms (Padua inventory) (*p *> 0.10). Similarly, the MIDI was not significantly impacted by participants’ age, education level, or gender (all *p *> 0.10). The MIDI has clinical relevance because it was associated with lower quality of life in participants, and furthermore, when participants returned to repeat the MIDI interview at least three months later, the concordance between time points was extremely high, supporting its test re-test validity but also the persisting nature of many impulsive disorders.

Using the MIDI, we found that 32.8% of the current sample had one or more impulsive disorders, the most common being gambling disorder. This was a somewhat enriched sample, because our study advertisements stated that the research was about ‘impulsive behaviors’ . Nonetheless, it is informative to compare the point prevalence rates observed here to those in other studies. The relatively low prevalence (<1%) of or kleptomania, trichotillomania, and pyromania, is in keeping with prior studies (Grant et al., 2014). A relatively high rate for gambling disorder (27.5% of our sample) was observed. In a meta-analysis of prevalence studies of gambling disorder amongst college students, the estimated proportion of pathological gamblers was 10.2% (Nowak and Aloe, 2014). We found that 5.9% of our sample met criteria for skin picking disorder on the MIDI. In a large sample of young adults in Poland, 7.7% of the sample had skin picking disorder (Prochwicz et al., 2016). Next most common in our sample were compulsive buying disorder (5.4% of the sample), compulsive sexual behavior (3.4%), intermittent explosive disorder (2.7%) and binge-eating disorder (2.7%). In a meta-analysis exploring compulsive buying, the overall prevalence estimate was 3.4–6.9%, being highest (8.3%) in college students (Maraz et al., 2016). The prevalence of compulsive sexual behavior was approximately 3–6% in older literature, and more recent work has found prevalence estimates of 2.0–3.7% in young adults using narrower definitions (Derbyshire and Grant, 2015). For intermittent explosive disorder, a current prevalence rate of 2.4% was found in a community sample (mean age 50 years, standard deviation 11.9 years), with aggression tending to peak in the third decade and diminish after the fifth (Coccaro et al., 2004). In meta-analysis, the 12-month prevalence of binge-eating disorder was average 0.9% (Qian et al., 2013). Viewed collectively, the current data are consistent with much of the available literature and suggest that many impulse control disorders are relatively common in young adults, but indicate that gambling disorder, skin picking disorder, and compulsive buying are particularly commonplace.

Several study limitations should be considered. As mentioned, the current sample was somewhat enriched for impulsive behaviors, and was conducted in young adults, and so these prevalence rates may not generalize to other populations (e.g. older participants, or young adults recruited using adverts not mentioning that the theme of the research was impulsivity). However, the mean scores on the Barratt impulsivenesss and Padua obsessive-compulsive scales were similar to normative data reported elsewhere, suggesting that our sample may be reasonably typical with respect to these measures (Burns et al., 1996, Spinella, 2007). On the other hand, prevalence rates for impulse control disorders (e.g. gambling disorder) were relatively high due to this being an enriched sample. Because our advertisements revealed the theme of the research, this may have resulted in respondents having recruitment and participant response bias. We could not evaluate the sensitivity and specificity of the MIDI against impulse control disorders other than gambling disorder, due to their relatively low prevalence; also, accepted gold-standard diagnostic instruments for these other disorders are largely lacking. We did not examine Cronbach's alpha for items within a given disorder, due to low frequency of multiple disorders; use of this measure at the level of the instrument would not have been appropriate because it evaluated multiple separate disorders. The term ‘impulse disorder’ was used for disorders in the MIDI but of course conditions sit in different diagnostic categories in DSM-5 (for example, gambling disorder is a Substance-Related and Addictive Disorder, whereas trichotillomania and picking are Obsessive-Compulsive Related Disorders, and binge-eating disorder is an Eating Disorder). On the Barratt questionnaire, attentional and non-planning but not motor subscores were significantly associated with a positive MIDI disorder; thus, attentional and non-planning aspects of impulsivity may be more rigorously associated across the broad swathe of disorders considered, with other variables being controlled for. Lastly, there is on-going debate about the optimal diagnostic criteria for some of the less well studied impulse control disorders; for pragmatic purposes, the MIDI uses similar criteria for each, except for gambling disorder (which is based on substance disorder criteria).

In summary, this study in an enriched sample of healthy adults supports the psychometric properties of the MIDI 2.0 as a useful tool for identifying and diagnosing a broad range of impulse control disorders based on current diagnostic criteria.

## Appendix A Minnesota Impulse control disorders Interview (MIDI) v2.0

Copyright the authors. Free for non-commercial academic use. Please contact Dr Grant for commercial use.

## Appendix B Supplementary materials

Image, application 1
